# Topological Properties of Atomic Lead Film with Honeycomb Structure

**DOI:** 10.1038/srep21723

**Published:** 2016-02-25

**Authors:** Y. H. Lu, D. Zhou, T. Wang, Shengyuan A. Yang, J. Z. Jiang

**Affiliations:** 1 School of Materials Science and Engineering, Zhejiang University, Hangzhou, 310027, China; 2 State Key Laboratory of Silicon Materials, Zhejiang University, Hangzhou 310027, China; 3 College of Electrical Engineering, Zhejiang University, Hangzhou, 310027, China; 4 Research Laboratory for Quantum Materials, Singapore University of Technology and Design, Singapore 487372, Singapore

## Abstract

Large bandgap is desired for the fundamental research as well as applications of topological insulators. Based on first-principles calculations, here we predict a new family of two-dimensional (2D) topological insulators in functionalized atomic lead films Pb-X (X = H, F, Cl, Br, I and SiH_3_). All of them have large bandgaps with the largest one above 1 eV, far beyond the recorded gap values and large enough for practical applications even at room temperature. Besides chemical functionalization, external strain can also effectively tune the bandgap while keeping the topological phase. Thus, the topological properties of these materials are quite robust, and as a result there exist 1D topological edge channels against backscattering. We further show that the 2D Pb structure can be encapsulated by SiO_2_ with very small lattice mismatch and still maintains its topological character. All these features make the 2D atomic Pb films a promising platform for fabricating novel topological electronic devices.

As a new class of quantum materials, topological insulators (TIs) have attracted a lot of attention because of their significant impact on the research of fundamental condensed matter physics and material science^1^,^2^,^3^,^4^. Topological insulators have an insulating bulk but spin-polarized gapless helical surface or edge states. Such gapless surface or edge states are protected by time-reversal symmetry and immune to scattering by nonmagnetic impurities or crystal defects. The study of TIs not only provides a new level of control over Dirac fermions, but may also lead to applications in low-power electronics and spintronics due to its unique transport properties against backscattering. For such application purposes, 2D TIs could be better than 3D TIs as the electrons in the topological edge channels of 2D TIs can only move along two opposite directions with opposite spin-polarizations hence backscattering is in-principle completely suppressed, whereas for the surface states of 3D TIs only the exact 180°-backscattering is suppressed but momentum relaxation through small-angle scattering is still possible. Many 3D TIs as well as their topological surface states were experimentally confirmed, while only HgTe/CdTe and InAs/GaSb quantum well systems have been well-established as 2D TIs by experiments so far^5^,^6^. Furthermore, the bulk-gaps of these quantum wells are so small that their topological edge states can be observed only below 10 K. It has thus been a long-sought goal to search for new 2D TIs^7^ which can possess a large bulk bandgap, hopefully ensuring its topological properties to manifest at room temperature.

The first proposed 2D TI is the carbon-based honeycomb lattice: graphene^8^. However, its bandgap is as small as 10^−4^ meV which cannot be probed by current experimental technologies^9^. Following graphene, a lot of other 2D honeycomb-structured materials have been investigated and predicted as 2D TIs, especially the 2D group IV honeycomb lattices^10^,^11^,^12^. For example, it is found that the spin-orbit coupling (SOC) gap of silicon with buckled honeycomb structure (known as silicene) is much larger than that of graphene and can be increased further by strain^10^. 2D Germanium with similar geometrical structure is also predicted as 2D TI with a gap larger than 20 meV^10^. Recently, Xu *et al.* predicted that the 2D functionalized Sn film is also a large-gap (~0.3 eV) 2D TI by first-principles calculations. Besides, some functionalized Bi, Sb and Pb bilayers also demonstrate 2D TI phases with large gaps that almost up to ~1 eV^13^,^14^,^15^,^16^.

The SOC gap size is related to the SOC strength of the involved elements: usually the heavier the element, the larger the SOC gap. The heaviest element of group-IV is Pb and the investigation of Pb’s topological properties is still scarce^16^. Here, we study the topological properties of 2D atomic Pb film with honeycomb lattice structure. It is found that both the planar and the low-buckled Pb films are 2D TIs with large gaps. Its gap size is sensitive to chemical functionalization and can be enhanced to around 1 eV by the decoration of hydrogen, halogen atoms or SiH_3_. Furthermore, we show that Pb films can be encapsulated by SiO_2_ while still maintaining its topological character. These results suggest 2D Pb films as promising topological materials for constructing low-power-consumption electronics which may operate even at room temperature.

## Results and Discussion

**Figure 1:**
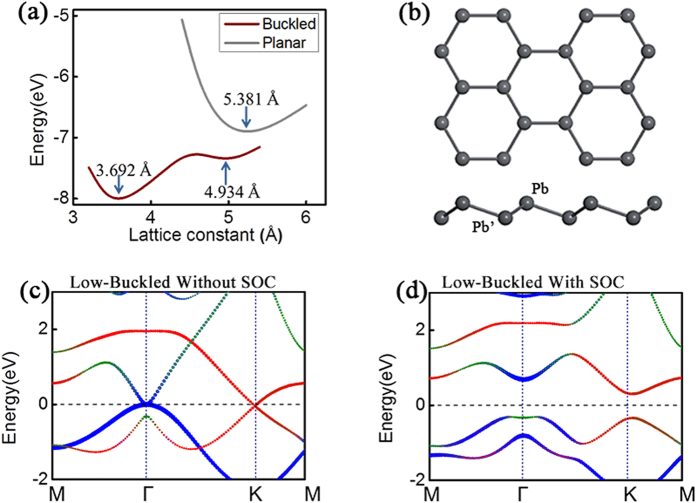
(**a**) Energy-Lattice constant relationship of Pb. The brown curve represents buckled monolayer while the grey curve represents plane monolayer of Pb. 3.692 Å, 4.934 Å and 5.381 Å mark out the lattice constants corresponding to the three local minimal values of the two energy curves. Here energy refers to the calculated total energy of unitcell. (**b**) Lattice structure of the low-buckled Pb layer from the top(upper) and side(lower) view. Pb is related to Pb’ by an inversion operation. (**c**,**d**) Band structure of 2D low-buckled Pb without & with SOC. The green, blue and red circles represent *s*, *p*_*x,y*_ and *p*_*z*_ orbital components respectively. The size of these circles is proportional to the contribution of the corresponding orbitals.

The honeycomb structure of carbon, graphene, has a planar structure because the *sp*_*2*_ hybridization is favourable for that system. Si and Ge prefer *sp*_*3*_-type bonding hence buckling is observed with a height difference between two sublattices. As shown in Fig. 1(a), energetically, Pb layers could have three stable structures, the lattice constants of these structures are 3.692 Å, 4.934 Å, and 5.381 Å respectively. Similar to the cases of Si and Ge^10^, the two buckled configurations with smaller lattice constants (3.692 Å, 4.934 Å) are more stable than the planar one. Pb is the most metallic element in group IV and the bonds between Pb atoms are somewhat different from the typical covalent bonds (*sp*_*2*_ or *sp*_*3*_) between other group IV elements. The most stable honeycomb structure of Pb (with lattice constant 3.692 Å) is high-buckled with a height difference of 2.55 Å, larger than that of silicene and germanene, and the next-neighbour bond length is very close to the neighbouring one, resulting in extra 6 Pb-Pb bonds in the structure. It is packed much closer than the covalent bonded Si and Ge and is similar to a metallic system. On the other hand, the low-buckled configuration with lattice constant 4.934 Å is found to be meta-stable. Figure 1(b) displays the optimized geometry of this low-buckled structure. The low-buckling enhances the π-π coupling between *p*-orbitals of Pb atoms in coexistence with certain overlap between π and σ orbitals. Actually, the competition between the π-π and π-σ couplings determines the buckled structure. The same mechanism also applies to silicene and germanene^17^. Both high and low buckled structures are more stable than planar geometry because *sp*_*2*_bonding is unfavourable between Pb atoms.

We calculated the band structures of these structures with and without SOC. The high-buckled structure is a metal without a bandgap, while both the low-buckled and the planar structures are insulators with a large bandgap after SOC is turned on. Considering the energetic stability and electronic properties, we mainly focus on the low-buckled one here. As shown in Fig. 1(c), in the absence of SOC, two energy bands cross linearly at the K point showing Dirac-cone-like features in the band structure, which is similar to that of graphene and other group IV 2D honeycomb lattices^12^,^18^. However, distinct from other group IV elements, the conduction band and the valence band here also touch at Γ point in the absence of SOC. By projecting onto atomic orbitals (see Fig. 1(c)), one observes that the low-energy bands around K point are mainly of *p*_*z*_ orbital character, while the low-energy bands around Γ point are dominated by *p*_*x*_*/p*_*y*_ orbitals. When SOC is turned on, the degeneracy at both the Dirac point (K point) and Γ point are lifted and gaps are opened at both points (see Fig. 1(d)). One observes several interesting features. First, like other group IV elements, the SOC gap opened at Dirac points and Γ point implies 2D TI phase, which is indeed confirmed by calculating the Z_2_ invariant through parity analysis. Second, the SOC opened bandgap is quite large. It is about 0.467 eV using the PBE functional. The gap value increases to as large as 0.612 eV by using hybrid functional. It is the largest one among the 2D materials made of group IV elements. Third, the local gap at Γ point is larger than that at K point, this is because of their different orbital character: at K point, the low-energy bands are from *p*_*z*_ orbitals which carries zero orbital angular momentum, hence SOC gap is from the first order next-nearest-neighbour hopping process through the coupling between *p*_*z*_ and *p*_*x*_*/p*_*y*_ orbitals of neighbouring atoms^14^; whereas at Γ point, the low-energy bands are from *p*_*x*_*/p*_*y*_ orbitals, hence the SOC gap directly derives from the first order on-site process^14^, leading to a larger local gap ~1 eV. Finally, due to the hybridization with the second valence band (from *p*_*z*_ orbitals) at Γ point, the valence band around Γ point is slightly above that at K point, resulting in a global indirect bandgap.

**Figure 2:**
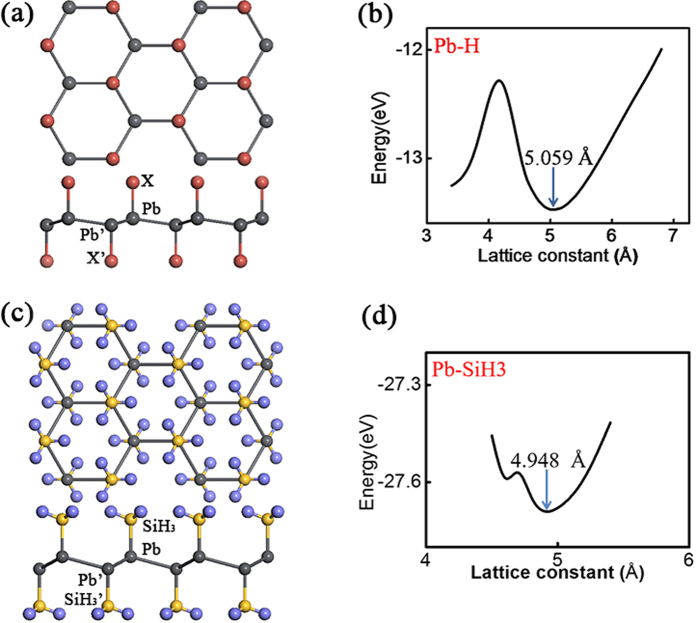
(**a**) Lattice structure of Pb-X layer from the top(upper) and side(lower) view. Pb-X is related to Pb’-X’ by an inversion operation. X represents the chemical functional group. (**b**) Relationship between lattice constants and the calculated total energy of Pb-H with most stable constant indicated. (**c**) Top and side view of Pb-SiH_3_, balls of yellow and blue represent silicon and hydrogen atoms respectively. (**d**) Relationship between lattice constants and the calculated total energy of Pb-SiH_3_ with most stable constant indicated.

**Figure 3:**
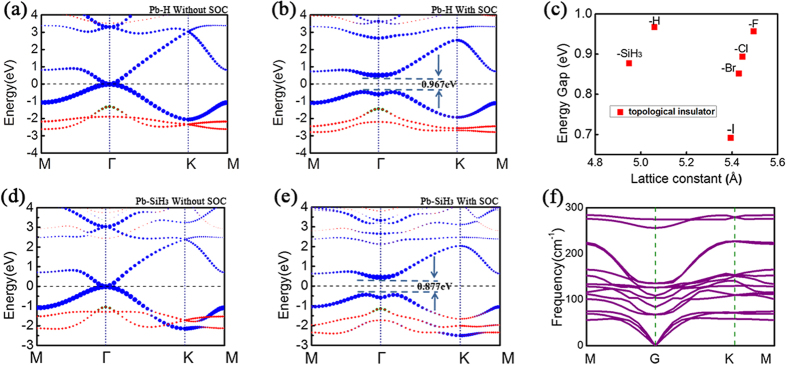
(**a,b**) Band structures of Pb-H without & with SOC. (**c**) The calculated global energy gap of Pb-X at their equilibrium lattice constants. All of them are 2D topological insulators. (**d,e**) band structures of Pb-SiH_3_ without & with SOC. The Fermi level is indicated by the dash line. The green, blue and red circles represent *s*, *p*_*x,y*_ and *p*_*z*_ orbitals separately, and the size of these circles is proportional to the contribution of the corresponding orbitals. (**f**). Phonon band dispersions of Pb-SiH_3_.

As demonstrated for graphene and stanene, chemical functionalization of 2D materials proves to be a useful tool to create new materials with specific features^19^,^20^. This provides us with a possible way for the designing of new TIs. Figure 2(a) present the lattice structure of Pb-X (X stands for F, Cl, Br, I and H). Figure 2(b) shows the total energy as a function of lattice constant of the Pb-H system. Due to the interaction between hydrogen and Pb film, the lattice constant of Pb buckled structure are slightly enlarged compared with the pristine one. We observe that the saturation of Pb *p*-orbitals make the low-buckled structure more stable. The low-buckled structure now becomes energetically more favourable than the high-buckled one. A stable *sp*_*3*_-like bonding configuration forms, which is similar to graphane. In Fig. 2(c), we show the optimized lattice structure for Pb-SiH_3_. Similarly, we find that the optimum lattice constant of Pb-SiH_3_ is also larger than the pristine one due to the chemical bonding between Pb and SiH_3_ group (Fig. 2(d)). More importantly, the electronic structures around Fermi level are modified by the functional groups. As an example, the band structure of Pb-H is shown in Fig. 3(a). One observes that a large gap opens at K point even without SOC because of the saturation of *p*_*z*_-orbital by hydrogenation. On the other hand, the conduction band minimum (CBM) still touches with the valance band maximum (VBM) at Γ point around the Fermi level, similar to the pristine one. This is because the bands around Γ are mainly composed of *p*_*x*_ and *p*_*y*_ orbitals and they are less affected by hydrogenation. When SOC is turned on, the conduction band and the valence band shift away from each other and a large gap opens. Comparison between Figs 1(d) and 3(b) shows that the effect of chemical functionalization: without hydrogenation, although the SOC gap at Γ point is as large as 1 eV, the global gap is limited by the smaller local gap at K point; with full hydrogenation, the *p*_*z*_ bands are pushed away from the Fermi level. As a result, the global bandgap would be entirely determined by the large SOC gap at Γ point. (We have also checked the half-hydrogenated case, which has a structure close to the high buckled one and shows metallic property (Fig. S5 in the Supplementary Information)). Similar physics apply for other functional groups as well. Particularly, the results for Pb-SiH_3_ are shown in Fig. 3(d,e). Without SOC, the VBM and CBM of Pb-SiH_3_ touch with each other at Γ point, whereas a giant local gap opens at K point. When SOC is considered, a gap of 0.877 eV opens at Γ point. We find that the global gaps of Pb-X (X = H, F, Cl, Br, I, and SiH_3_) layers range from 0.692 to 0.967 eV at PBE level (the gap values are shown in Fig. 3(c)). In retrospect, we can see that such large gap values are because of three reasons: (1) Pb atoms have strong atomic SOC strength due to the large atomic number; (2) the states at Γ point around Fermi level have *p*_*x*_*/p*_*y*_ orbital character hence the SOC is of on-site type with a large strength comparable to the atomic SOC of Pb^15^; (3) chemical functionalization pushes the *p*_*z*_ bands away from low energy, exposing the SOC gap around Γ point as the global bandgap. The band topologies of all our systems are again confirmed by the parity analysis at the four time reversal invariant momenta (one Γ point and three M points)^21^. We find that all these systems are topologically nontrivial with non-zero Z_2_ numbers. Thus, functionalized Pb monolayer structures are large bandgap 2D TIs. Moreover, we point out that the gap values in Fig. 3(c) are still underestimated due to the well-known problem of PBE functional. For example, the global bandgap of Pb-H would be increased to 1.141 eV when hybrid functional is used (see Fig. S4 of SI). As far as we know, this is the largest bulk band gap of all reported 2D TIs. The underlying physical mechanism is similar to that of the functionalized stanene and methyl-functionalized Pb^12^,^16^. It is noted that the hydrogenated Sn (or called stanane) is a trivial system^12^, different from the topological character of hydrogenated Pb (Pb-H) system studied here, although the geometrical structures of them are very similar. The difference could be attributed to two reasons: Firstly, the band order at Γ point of Pb-H without SOC is different from that of stanane. The *s* orbital of stanane at Γ point is above both *p* orbitals and Fermi level^12^, while for the Pb-H, *s* orbital is lower in energy than *p* orbitals and Fermi levels (Fig.3(a)), indicating the topological difference between them without SOC^22^. Secondly, smaller SOC strength in Sn compared with Pb is not sufficient to invert the bandgap for Sn-H around Γ point.

**Figure 4:**
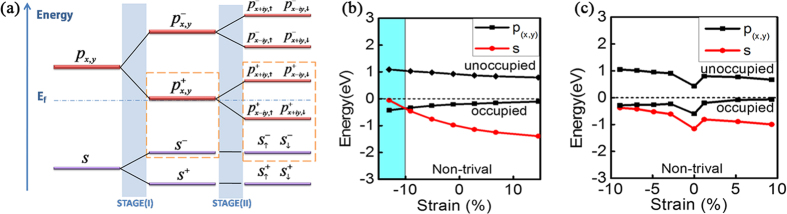
(**a**) The schematic diagram of the orbital evolution at the Γ point for Pb-H. The stage (**I**) and (**II**) represent the effect of chemical bonding and spin-orbital coupling. The blue dot-dash line represents the Fermi level. (**b,c**). Evolution of *s* and *p*_*x,y*_ orbitals at Γ point under hydrostatic strains of Pb-H and Pb-SiH_3_ respectively. The dashed line represented the Fermi level, and light blue shaded area highlights the topologically trivial region.

To further understand the nature of large SOC gap of Pb-X system, we take the hydrogenated Pb layer (Pb-H) as an example to analyse the evolution of low-energy states around the Γ point. Figure 4(a) illustrates the two stages (I and II) of orbital splitting. Based on the first principle calculations, initially, the low-lying bands around Γ point are mainly composed of *s* and *p*_*x,y*_ orbitals from Pb atoms (6 s^2^ 6p^2^) whose *p*_*z*_ orbital is compensated by H (1 s^1^). During stage I, the chemical bonding between Pb atoms will form bonding and antibonding states for both *s* and *p*_*x,y*_ orbitals. These states are labelled as “ ± ” indicating the parity. Before turning on SOC, the material is a semi-metal, since the *p*_*x*_ and *p*_*y*_ bonding states are degenerate at the Fermi level because of the C_3_ rotational symmetry. With the SOC effect considered in stage II, the degeneracy of them is lifted and open a large SOC gap^22^ and whole system becomes a nontrivial topological insulator.

We then investigate the effects of strain on the topological properties of these 2D TIs. We plot the energy levels at Γ point of Pb-H and Pb-SiH_3_ layers, under different hydrostatic strains in Fig. 4(b,c). One observes that the antibonding state from the *s* orbital shifts upward with respect to the bonding state of *p*_*x,y*_ orbitals under a compressive strain. And for Pb-H, a crossing between the *s* level and the *p*_*x,y*_ level appears at a critical strain value around 10%. This leads to a topological phase transition during which the nontrivial Pb-H transforms to a trivial insulator. On the other hand, the topological nontrivial state remains under tensile strain. All other Pb-X systems show similar behaviours under strain. For Pb-SiH_3,_ topological nontrival properties remain for both compressive and tensile strains up to 10%. Therefore, we can see that the topological nontrivial properties are robust against moderate strains. In addition, the nontrivial bandgap can be tuned by strain or by choosing different functional groups (Fig. 3(c)). Experimentally, strain can be applied, e.g., by putting the material onto a flexible substrate and then bending/stretching the substrate, or by depositing the material onto a piezoelectric material and using electrical means to control the strain^23^,^24^.

**Figure 5:**
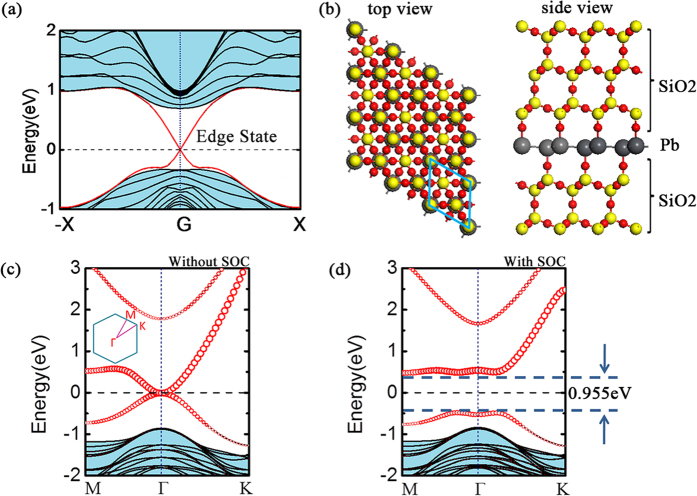
(**a**) Band structure of armchair-edged nanoribbons for Pb-H buckled monolayer. The ribbon width is 5.5 nm. Helical edge states are visualized by red lines crossing linearly at the Γ point, the light blue shaded area represents the bulk bands. (**b**) Top and side view of 3D structure of SiO_2_-Pb-SiO_2_ sandwich system, which contains Pb (grey balls), Si (yellow balls) and O (red balls). The blue rhombic in top view shows the primitive unit cell. (**c,d**) Band structures of the SiO_2_-Pb-SiO_2_ sandwich system without and with SOC. Size of the red circles is proportional to the contribution of the p orbital of lead atoms, dashed lines represent the Fermi level, the blue shaded area represents the bands of SiO_2_. The K-path in 1^st^ Brillouin zone of this system is shown in the inset of (**c**).

The unique character of 2D TI is the gapless edge states which are helical with spin-momentum locking. The edge states act as perfect 1D conducting channels without scattering by non-magnetic defects. To explicitly show the edge states, we perform a calculation of a Pb-H nanoribbon structure. Figure 5(a) shows the result of a ribbon with armchair-type edges and with H termination. The results are qualitatively the same for other types of edges. Here the width of the nanoribbons is large enough to avoid the interaction between the two edges. One clearly observes the gapless edge states crossing within bulk gap. A single pair of helical edge states exists on each edge, in agreement with the Z_2_ topological character obtained from the parity analysis. These helical edge states are useful for low-dissipation electronics and spintronics devices because of their robustness against scattering.

We further investigate the dynamical stability of these 2D layers by calculating their phonon spectra. We find that the Pb-SiH_3_ system is dynamically stable without any soft mode in the phonon spectrum (Fig. 3(f)). Meanwhile, for other functional groups, there exist imaginary frequencies, implying that these 2D layers may not be stable in the free-standing form. Their stabilization would require suitable substrates or encapsulation. Indeed, for real applications, the 2D layered structure also needs to be put onto suitable substrates when fabricated. In the following, we show that the SiO_2_ substrate could be used as an excellent encapsulation layer for the 2D Pb film, which maintains the nontrivial topological properties of the Pb layer. So far, most electronic devices and circuit are fabricated on silicon surfaces and the silicon wafer is the most popular substrate. Normally, the silicon surface is oxidized and covered by SiO_2_ and crystalline SiO_2_ can be also obtained in well-controlled experiment^25^. Here, we investigate 2D Pb film encapsulated by β-tridymite SiO_2_ substrates (with space group P63/mmc). The geometrical structure is shown in Fig. 5(b). We find that the lattice mismatch between them is very little, only ~2.7%, indicating the feasibility of fabrication in experiment. The sandwiched Pb atoms are bonded with oxygen atoms and the whole film remains a low buckled honeycomb structure with a lattice constant matched to β-tridymite SiO_2_ of 5.063 Å. Figure 5(c,d) present the calculated band structures without and with SOC effect, where red dots denote the states form the Pb layer. The band opening scenario due to SOC is almost the same as that of Pb-H and the gap value is as large as 0.955 eV at PBE level. As we discussed above, the 2D TI phase of the Pb-H honeycomb structure remain under a range of stain, hence moderate mismatch between the Pb layer and the substrate hardly change its topological character. The topological properties of such sandwiched structure are also confirmed by parity analysis. Our results therefore demonstrate that 2D Pb honeycomb film with nontrivial topological phase could be integrated into silicon systems which are widely used in current industry. It is noted that the similar sandwich structure was proposed as a method to enhance the SOC gap in graphene through proximity effect^26^,^27^,^28^,^29^. However, the SOC gaps induced for graphene are still much smaller than Pb-based system discussed here due to the very weak SOC from the carbon atoms.

In summary, we found a series of new large-gap 2D TI materials: Pb-X (X = F, Cl, Br, I, H, and SiH_3_), which have large bulk bandgaps on the order of 1 eV that is far beyond the existing experimentally realized 2D TI materials. These large values of gaps are due to the strong SOC effect caused by the heavy Pb atoms as well as the *p*_*x*_/*p*_*y*_ orbital character of the low-energy states. We show that the topological properties are robust against moderate strains. Both functional groups and the external strains can be used to effectively tune the electronic properties of the Pb layer. We explicitly demonstrate the gapless topological edge states on the edge of a nanoribbon. We find that Pb-SiH_3_ is dynamically stable without any soft phonon mode. The 2D layers with other functional groups may not be stable in the free-standing form and suitable substrate/encapsulation will be needed. Particularly, we find that the SiO_2_ offers an excellent encapsulation layer. We show that the large-gap 2D TI phase is maintained with a very small lattice mismatch to the SiO_2_ encapsulation layer in a sandwich structure. Due to their large gaps, our predicted 2D TIs could be probed even at room temperature. And other intriguing topological phases/objects such as quantum anomalous Hall phase and Majorana fermion modes may also be engineered based on these systems by suitable doping or interfacing with superconductors etc. As for the material synthesis, it could be possible to first grow a monolayer Pb by MBE on suitable substrates like hexagonal boron nitride or HOPG, then chemical functionalization may be achieved by exposure to atomic or molecular gases or by chemical reaction in solvents. In view of the rapid progress in the experimental techniques, the proposed structures are likely to be realized in the near future. Our discovery therefore points out a promising new material platform for both fundamental research as well as topological quantum device applications.

## Methods

In this paper the ab-initio density functional theory (DFT) calculation is used to investigate the material’s electronic and structural properties^30^. The calculation is carried out by using the projector-augmented-wave pseudo potential method^31^ with a plane wave basis set and the cutoff energy is set above 400 eV. Besides, the exchange correlation functional was treated by Perdew-Burke-Ernzerhof (PBE) generalized gradient approximation^32^. The k-mesh was sampled by Gamma point centred methodology with a grid size of 9 × 9 × 1. Thickness of the vacuum region is set to be 20 Å to avoid interaction between each layer and their periodic images. All the lattice constants and atomic positions are relaxed until the maximal force on each atom was smaller than 0.01 eV/Å. The electronic structures and topological properties were further verified by calculations using the hybrid functional^33^. For films in TI states, the two types of functionals predict the same nontrivial character. CASTEP code is used to calculate the phonon dispersion^34^.

## Additional Information

**How to cite this article**: Lu, Y. H. *et al.* Topological Properties of Atomic Lead Film with Honeycomb Structure. *Sci. Rep.* **6**, 21723; doi: 10.1038/srep21723 (2016).

## Supplementary Material

Supplementary Information
