# Supplementary material for: Topological Properties of Atomic Lead Film with Honeycomb Structure
Source: Sci Rep. 2016 Feb 25;6:21723. doi: 10.1038/srep21723 (PMC4766466; doi:10.1038/srep21723)

Topological Properties of Atomic Lead Film with Honeycomb Structure

Y.H. Lu,[1,2] D. Zhou,[1,2] T. Wang,[3,*] Shengyuan A. Yang[4,*], and J.Z. Jiang[1,2]

1. School of Materials Science and Engineering, Zhejiang University, Hangzhou, 310027, China.

2. State Key Laboratory of Silicon Materials, Zhejiang University, Hangzhou 310027, China

3. College of Electrical Engineering, Zhejiang University, Hangzhou, 310027, China.

4. Research Laboratory for Quantum Materials, Singapore University of Technology and Design, Singapore 487372, Singapore.

Fig.S1 (a), (b) Band structures of Pb with lattice parameters of 3.692 Å and 5.381 Å. Red solid lines (black dash lines) represent the band structure with (without) spin-orbit coupling. Dotted line represents the Fermi level.

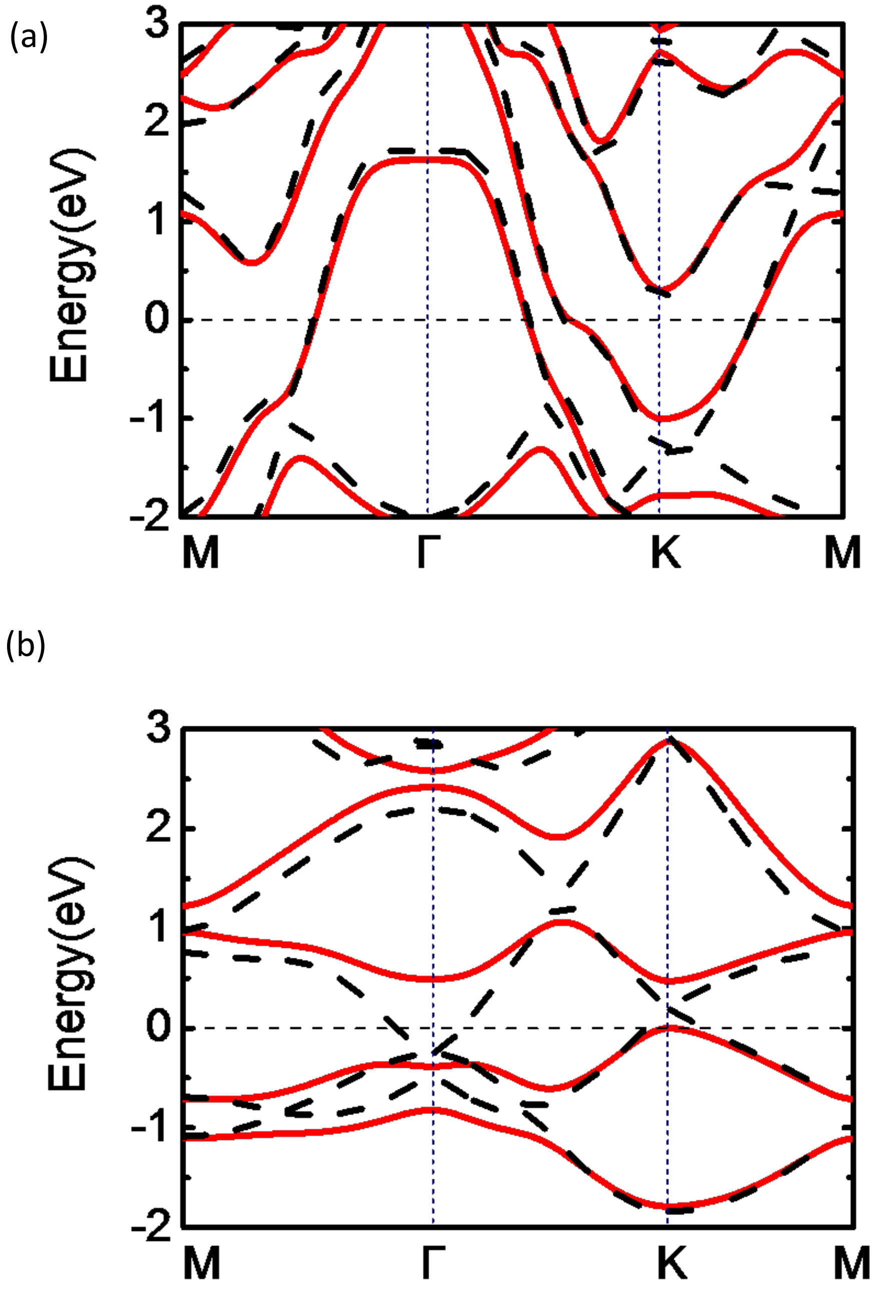

Fig.S2 (a), (b), (c), (d) Relationships between lattice constants and the calculated total energy of Pb-F, Pb-Cl, Pb-Br and Pb-I respectively. The equilibrium lattice constants of them are 5.495 Å, 5.446 Å, 5.430 Å and 5.394 Å respectively.

(a)

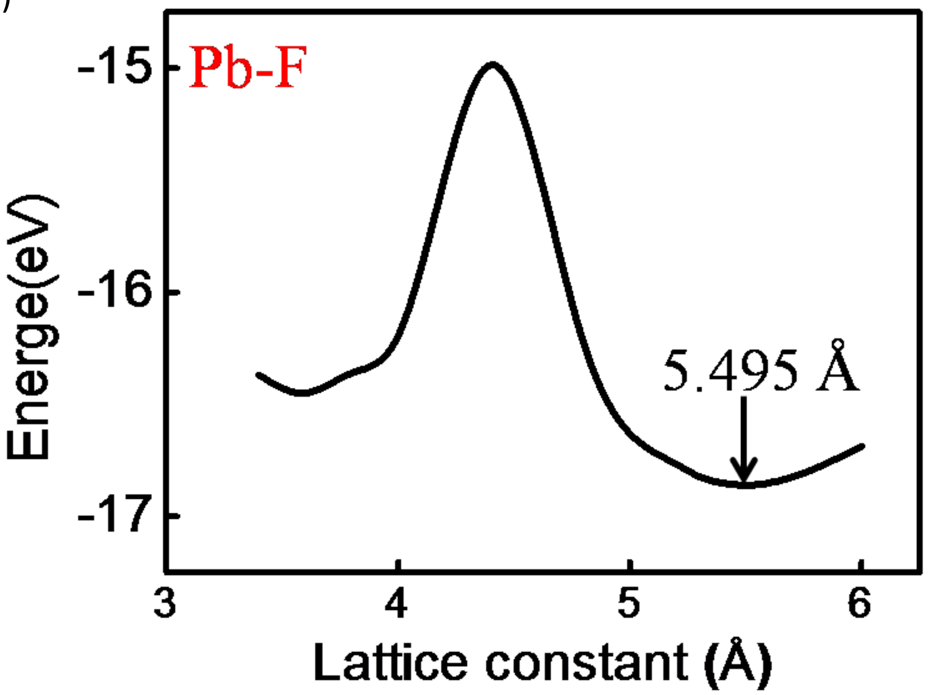

(b)

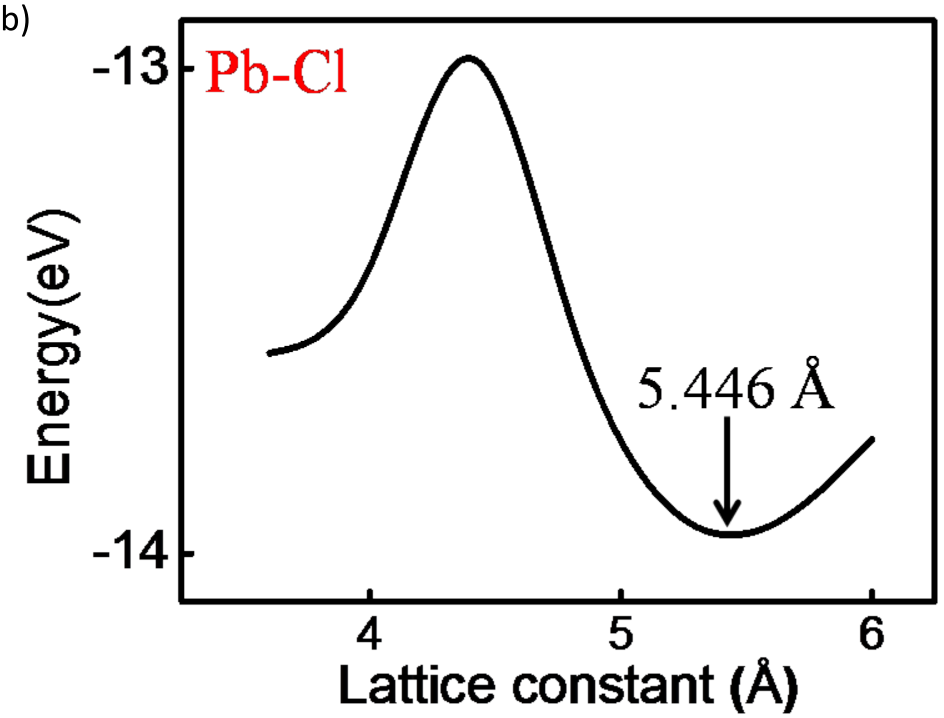

(c)

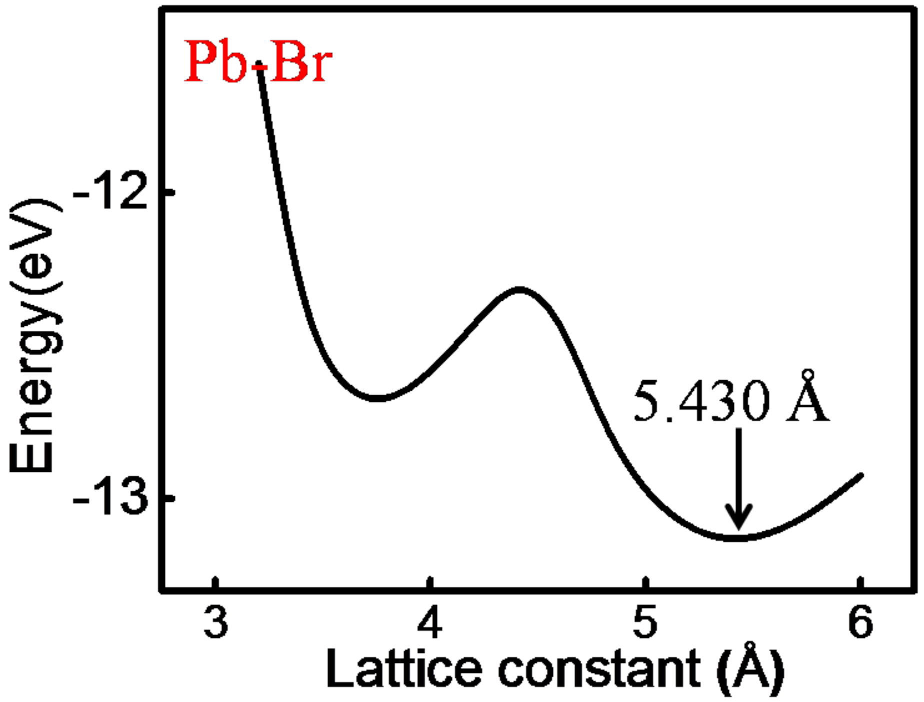
Pb-Br
-12
-13
Energy(eV)
5.430 Å
3
4
5
6
Lattice constant (Å)

(d)

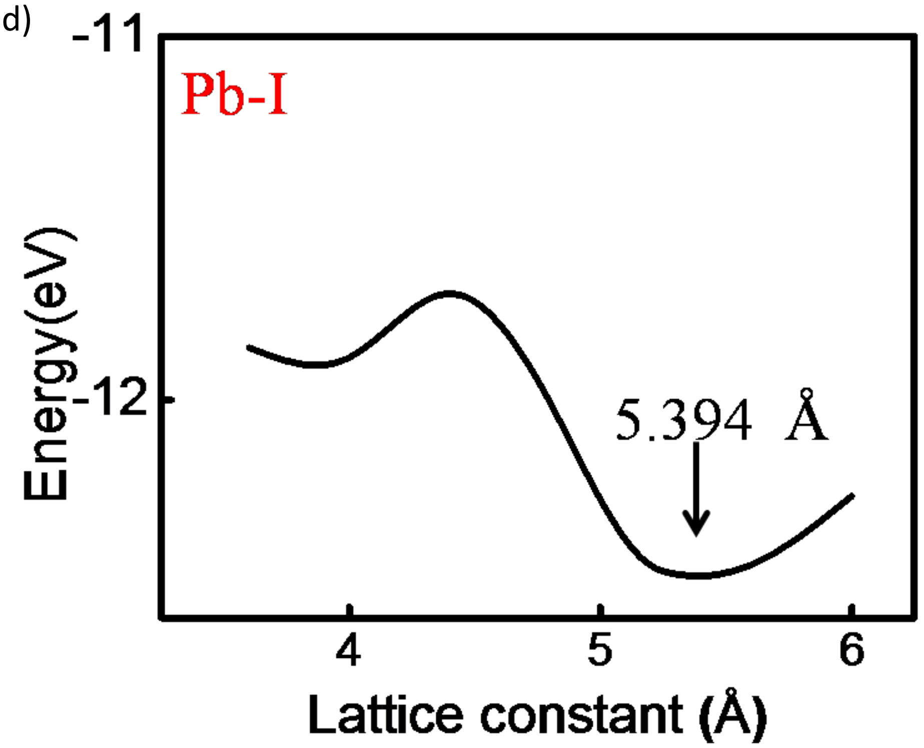
-11
Pb-I
-12
Energy(eV)
5.394 Å
4
5
6
Lattice constant (Å)

Fig.S3 (a), (b), (c), (d) Band structures of Pb-F, Pb-Cl, Pb-Br, Pb-I with (red solid line) and without (black dashed line) SOC. The Fermi level is indicated by the dotted line. The values of gap at the Γ point are 1.080eV, 0.995eV, 0.960eV and 0.911eV; the values of globe gaps are 0.957eV, 0.894eV, 0.852eV and 0.692eV.

(a)

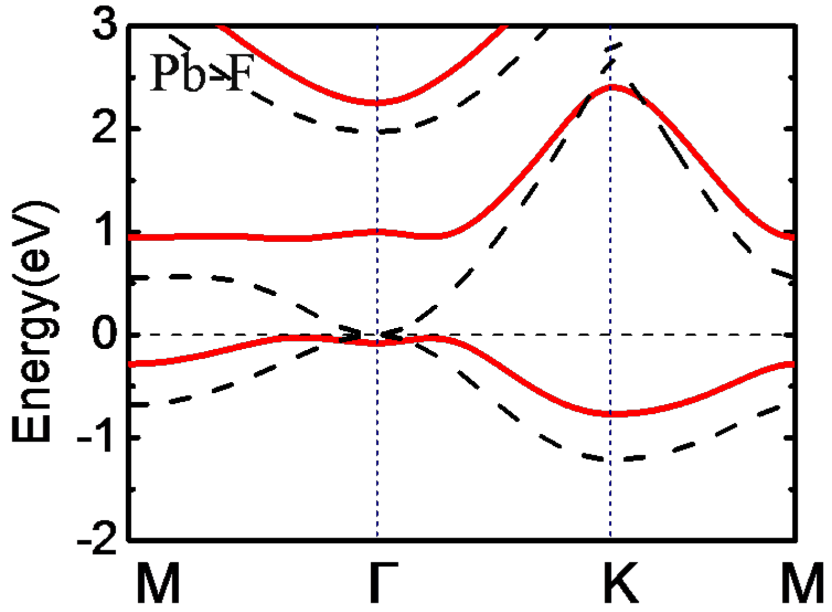

(b)

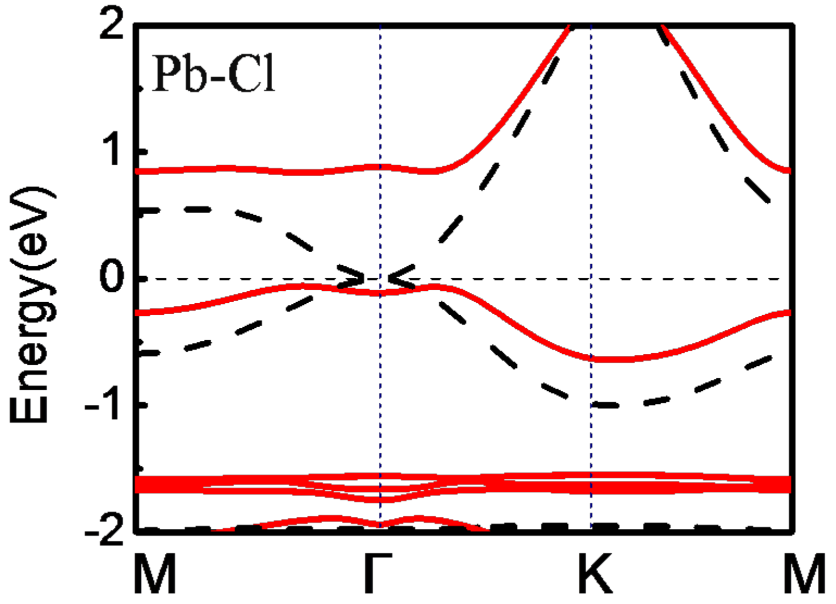

(c)

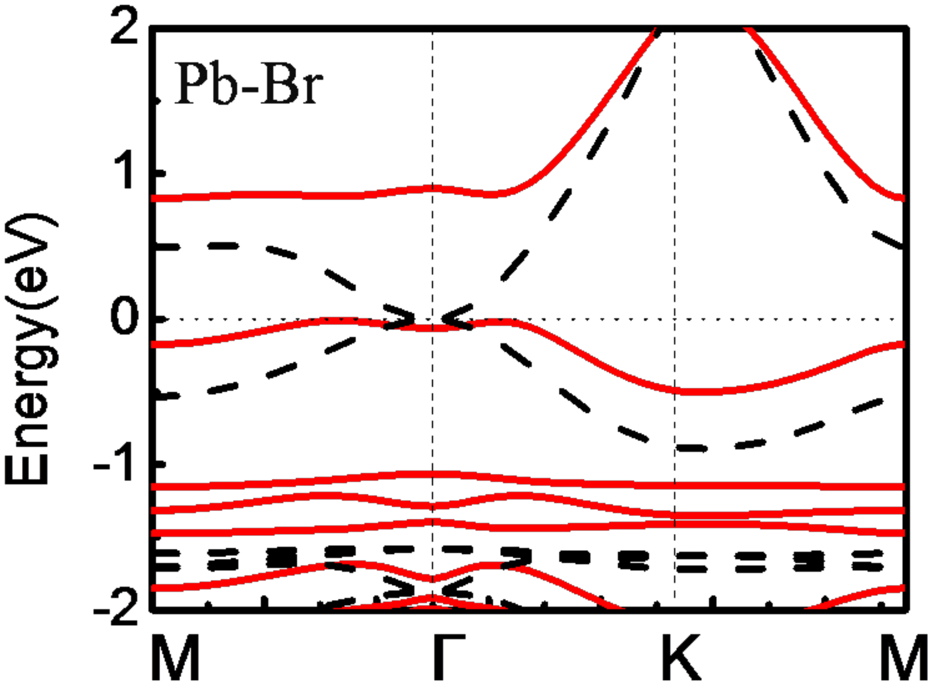

(d)

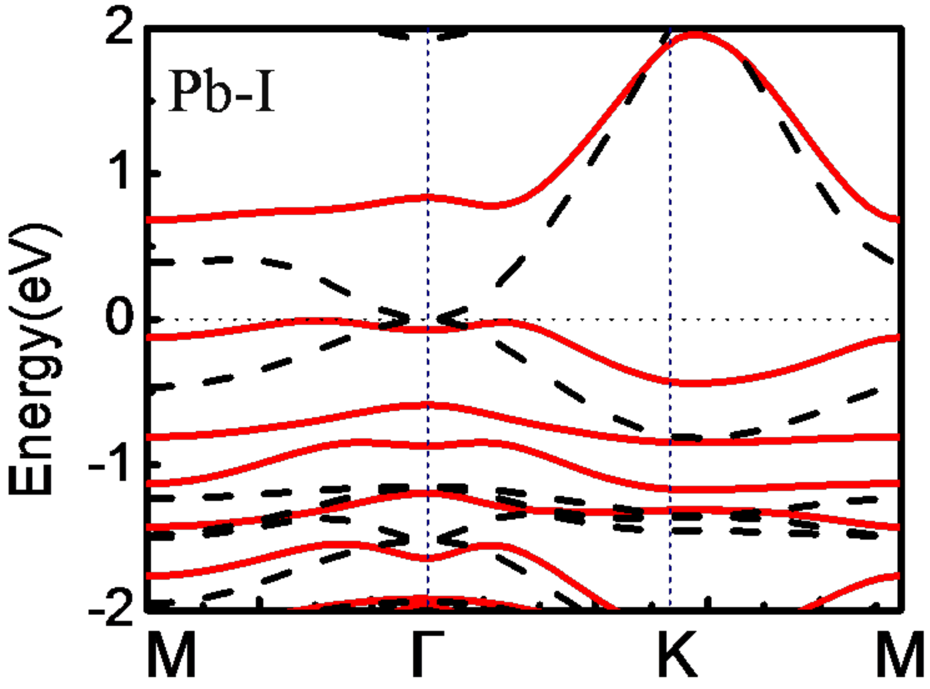

Fig.S4 Band structures of (a).Pb and (b).Pb-H using hybrid functional (HSE06) with (red solid) and without SOC (black dash).

(a)

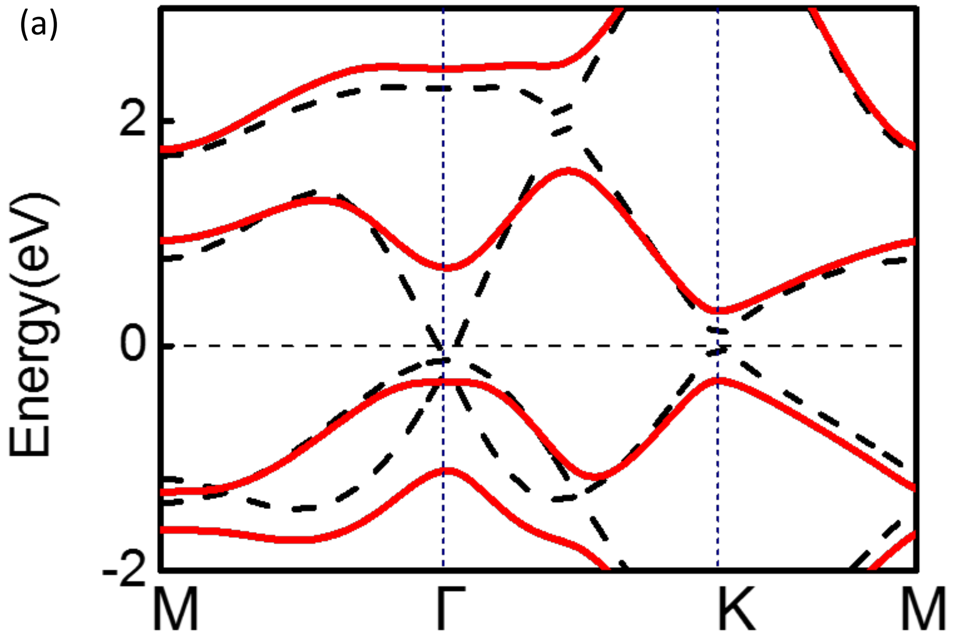

(b)

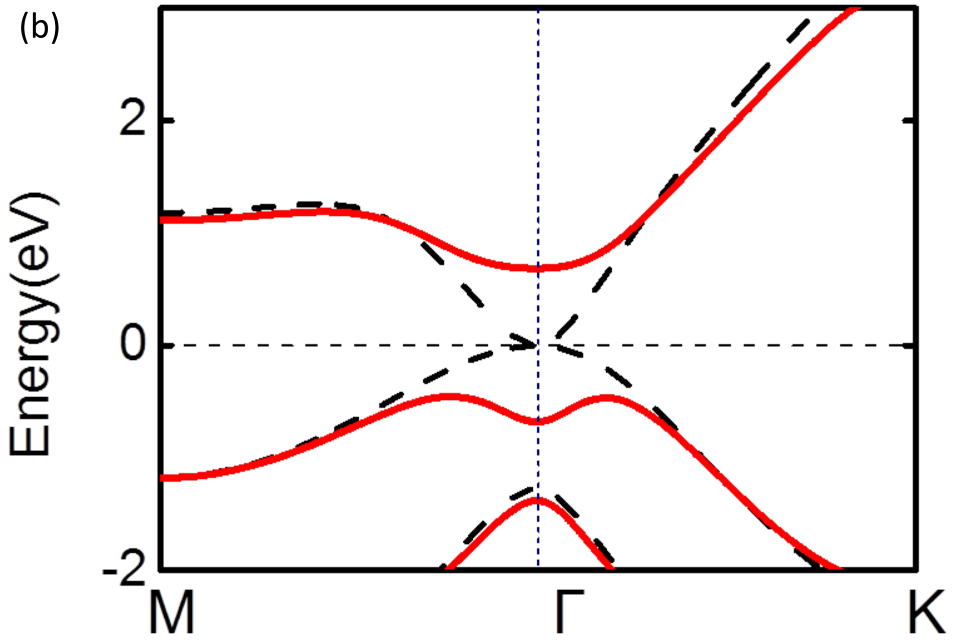

Fig.S5 (a). Band structures of half hydrogenated Pb without SOC. Spin up and spin down are represented by grey lines and black lines respectively. (b). Band structure of half hydrogenated Pb with SOC.

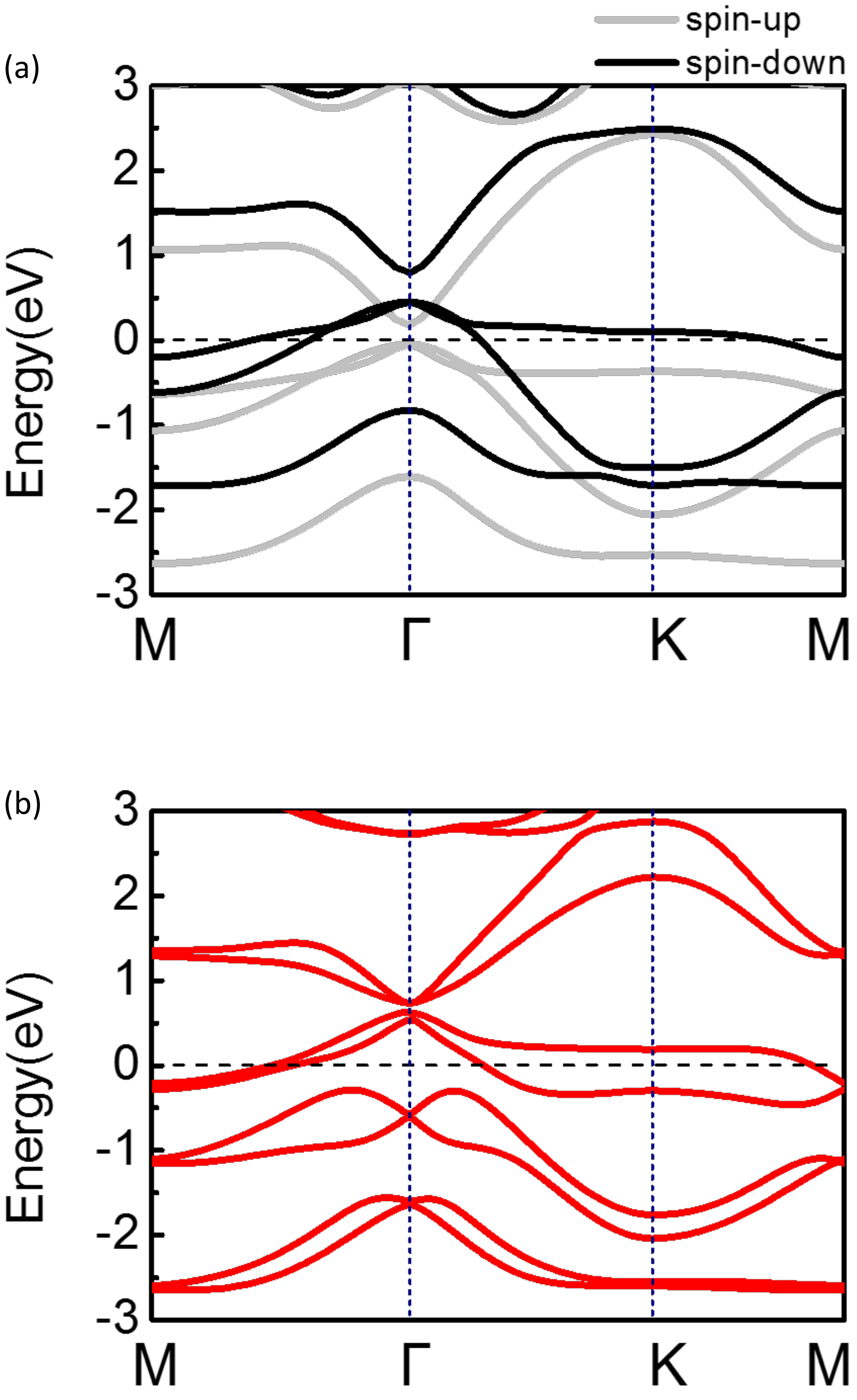

Supplement: Supplementary Information [file srep21723-s1.pdf]
